# Use of an objective measure of time to recovery after cardiac surgery – the STET randomised controlled trial

**DOI:** 10.1186/1745-6215-12-S1-A68

**Published:** 2011-12-13

**Authors:** Chris A Rogers, Katie Pike, Gianni D Angelini, Barney C Reeves

**Affiliations:** 1 Bristol Heart Institute, University of Bristol, Bristol, BS2 8HW, UK

## Objective

The STET trial is a two-centre open RCT comparing morbidity and healthcare resource use when off-pump coronary artery bypass surgery is carried out via a left anterolateral thoracotomy incision (ThoraCAB) or via a conventional median sternotomy incision (OPCAB). It was hypothesised that post-operative recovery would be faster with ThoraCAB.

## Methods

Post-operative hospital stay is a commonly used measure of recovery. However, in an open surgical trial, where the decision to discharge the patient from hospital lies with the surgeon, post operative hospital stay is susceptible to bias. For the STET trial we developed an objective measure of recovery. Recovery time was defined as the time from surgery until the patient was considered fit for discharge. Patients were classified fit (a) when the x-ray was clear (no evidence of pleural effusion requiring drainage, lung collapse/consolidation, pneumothorax); (b) there was no suspected systemic, lower respiratory tract or wound infection; (c) routine blood results and temperature were normal and (d) when physically mobile (walking 70m, bowels open and oxygen saturation>95%). These recovery criteria were monitored daily until discharge.

## Results

184 patients were recruited (91 randomised to ThoraCAB, 93 to OPCAB). In the OPCAB group 77% were classified fit at or before discharge versus 68% in the ThoraCAB group. For the remainder, the recovery time was censored because discharge occurred before all the criteria were met. Insufficient mobility accounted for the majority of censored observations. The median recovery time was 6 days, IQR [4,7] in the ThoraCAB group versus 5 days, IQR [4,7] in the OPCAB group (Time ratio (ThoraCAB/OPCAB) 1.03 (95%CI [0.94, 1.14], p=0.53). In contrast, the median time to discharge was 5 days in the ThoraCAB group versus 6 days in the OPCAB group.

## Conclusion

The anticipated faster recovery with ThoraCAB was not found and a significant proportion of patients were discharged before all the recovery criteria were met. The results from the STET trial illustrate the bias associated with clinical decision making in an open RCT. The measure of recovery time (with slightly modified criteria) is being used in other cardiac surgery trials.

